# COVID-19 mortality rate prediction for India using statistical neural networks and gaussian process regression model

**DOI:** 10.4314/ahs.v21i1.26

**Published:** 2021-03

**Authors:** S Dhamodharavadhani, R Rathipriya

**Affiliations:** Department of Computer Science, Periyar University, Salem-India

**Keywords:** Covid-19, India, mortality rate, mortality prediction, regression model, hyperparameter tuning, GPR, GRNN, RBFNN

## Abstract

The primary purpose of this research is to identify the best COVID-19 mortality model for India using regression models and is to estimate the future COVID-19 mortality rate for India. Specifically, Statistical Neural Networks (Radial Basis Function Neural Network (RBFNN), Generalized Regression Neural Network (GRNN)), and Gaussian Process Regression (GPR) are applied to develop the COVID-19 Mortality Rate Prediction (MRP) model for India. For that purpose, there are two types of dataset used in this study: One is COVID-19 Death cases, a Time Series Data and the other is COVID-19 Confirmed Case and Death Cases where Death case is dependent variable and the Confirmed case is an independent variable. Hyperparameter optimization or tuning is used in these regression models, which is the process of identifying a set of optimal hyperparameters for any learning process with minimal error. Here, sigma (σ) is a hyperparameter whose value is used to constrain the learning process of the above models with minimum Root Mean Squared Error (RMSE). The performance of the models is evaluated using the RMSE and 'R2 values, which shows that the GRP model performs better than the GRNN and RBFNN.

## Introduction

At the end of December 2019 in Wuhan, China, it was first reported that a human infection was caused by a novel coronavirus (nCov) or Wuhan virus or 2019-nCov^1^. One of the biggest challenges of this epidemic is a human-to-human transition of nCov. The coronavirus (COVID-19) infected cases increase at an exponential rate worldwide. On 30 January 2020, the World Health Organization (WHO) issued a worldwide health emergency warning notice 2, describing that 2019-nCoV is of critical global concern. The morbidity and mortality rates for the COVID -19 are uncertain at the early stage 3 especially for young ones and aged people. WHO has estimated the reproduction factor (R0) of nCov is 2.7. To monitor the massive and rapid spread of the nCov, public health sectors took reliable preventative measures. They imposed curfew or lockdown infested cities in China, the United States, India, and other countries also. This is to limit the social distance between people and to avoid the spread of this novel virus via humans to humans.

For the last decade, machine learning techniques have gain momentum and play a vital role in many domains of research fields. Notably, it has a tremendous impact on data analytics and data science. It enables a better understanding of the data and its methods, allows for future assumptions based on past data / empirical data, and automatically classifies the type of data (known as classification). Machine Learning (ML) techniques also can be used to develop standard mortality models.

Generally, ML tools used for prediction of the mortality rate of epidemic diseases in advance which helps the public healthcare authorities to develop and design an effective and efficient plan to reduce deaths. Authors Deprez et al. 4, used machine learning algorithms to fit and assess the mortality model by detecting the weaknesses of different mortality models. Artificial Neural Networks (ANNs)^5^ used to identify and forecast latent mortality variables with higher predictive accuracy. In 6, the authors used neural networks to expand the Lee-Carter model to several predictions of populations. Gaussian process models have typically been used extensively in engineering-based optimization applications (Razavi et al. 7). A combination of GPR and adaptive neuro-fuzzy inference system (ANFIS) 8 used in groundwater level forecasting. In 9, an extensive comparative study was carried out between several surrogate models, comprising GPR, using simulation-optimization methodology with uncertainty parameters. In the end, they had concluded that the GPR models and their ensemble were efficient methods concerning prediction accuracy. GRNN model was built in 10 as a new computational method for the field of incidence prediction of infectious diseases. Han, et al. 11 developed a GRNN network with a one-dimensional input and output layer to predict blood, and sexually transmitted infections are occurring. In 12, ―authors implemented a comparison analysis on Back Propagation Neural Network (BPNN), Generalized Regression Neural Network (GRNN), and Radial Basis Function Neural Network (RBFNN) network for prediction of the evaporation‖. The results showed that the GPR is a successful technique compared with artificial neural network approaches.

In^13^, ―a large-scale comparison analysis was presented for major machine learning models such as multilayer perceptron, Bayesian neural networks, radial base functions, generalized neural regression networks (also kernel regression), K-nearest neighbor regression, CART regression trees, support vector regression, and Gaussian time series prediction processes‖. The authors observed that the performance of these models was solely dependent on the data set, having different impacts.

The RBF and GRNN 14 were applied to data of patients with heart disease for the medication outcome. The results showed that RBF performed well for prescribing medicine for the patient. In 15, the authors claimed that the Gaussian process approach performed better than the standard generalized linear model (GLM) for the Phenomenological forecasting of dengue disease incidence. Gholam Ali Montazer et. al 16 reviewed various learning methods for defining network parameters such as widths, centers, and synaptic weights of the RBF neural network. In 17, extensive neural regression networks were proposed as an automated technique for forecasting time series. This technique is intended to achieve an efficient and fast tool for automatically predicting a vast amount of time series. From these works, one could clearly understand the applications of GPR, GRNN, and RDFNN in various research domains.

In this work, the GPR model with optimized hyperparameter (Co-variance, mean), GRNN with optimized hyperparameter (spread), and RBFNN with optimized hyperparameter (spread) are applied to develop COVID -19 mortality models for two types of dataset. Moreover, to evaluate the performance of these models, RMSE, a quantifiable measure will be used, and the MRP model with low RMSE will be selected as the best model for predicting the COVID-19 mortality rate for India. The purpose of this study is to predict mortality rates against multiple COVID-19 confirmed cases using machine learning techniques that capture patterns that cannot be identified by a standard statistical mortality model.

The rest of the paper is organized as follows. Section 2 details the methods and materials for Covid-19 mortality rate forecasting for India. The outcomes and analysis of this study are given in Section 3. This work is summarized in Section 4 with potential future research.

## Methods and materials

### A Hyperparameter Optimization

The goal of hyperparameter optimization in regression models is to find the parameters of a given regression technique, which returns the best output on a validation set while training and testing the model^15^. This is as shown in equation (1)

(1) $$\text{H}*={\text{arg min}}_{\text{o}\in \text{O}}\text{f(o)}$$

Where f(O) is an objective score to minimize RMSE calculated on the validation dataset; H * is the set of hyperparameters that gives the lowest RMSE score, and o is any value in the problem domain O.

Even though hyperparameter optimization is costly in terms of computational time, it yields good prediction accuracy than traditional regression models.

### B Gaussian Process Regression

Gaussiaprocessss is a machine learning technique used to make uncertain predictions. It is defined as a finite set of random variables distributed jointly by the Gaussians 15. These random variables represent the value for a function f(x) at input x in regression problems. It is represented as {f(x) : x X} defined by the mean function μ(x) and the covariance function k(x, x′) so it can be represented as

(2) f(⋅)~GP(μ(⋅),k(⋅,⋅))

Usually, a zero-mean Gaussian process before equation (2) is the prior distribution over functions f (•) Same detailed explanation found in 16. This model is named, in literature, a substitute for the objective function. The substitute can be configured more easily than the intent feature. A GP method determines the next set of hyperparameters to test by choosing the best hyperparameters acting on this surrogate function on the actual objective function.

A covariance function as defined in equation (3) is used to represents the covariance between pairs of random variables in GPR.

(3) $$K_{ij}=k(x_{i},x_{j})=\alpha \exp\{\frac{||x_{i}-x_{j}|{|}^{2}}{2\sigma_{1}^{2}}\}$$

Here, hyperparameters are

*σ*_1_ = *Characteristic lengthscale*

*α = Signal variance*

**GPR model-based hyperparameter optimization**

*1. Initialize hyperparameters for GPR model*

*2. Define an objective function of GPR model*

*3. Specify the selection criteria (ie. Minimal RMSE) for evaluating hyperparameters which have to choose next from the surrogate model*

*4. Update the surrogate model by (score, hyperparameter) pair*

*5. Repeat steps 2-4 until maximum iterations or time is reached.*

### C GRNN

A special case of Radial Basis Networks (RBN) is the Generalized Regression Neural Network (GRNN) 28. The structure of a GRNN with two layers is comparatively simple and fixed. The first is the sequence, and the second is a summation. When the input is passed through each unit in the pattern layer, the input-response relationship will be “memorized” and stored within the unit. As a result, the number of units in the pattern layer equals the number of individual values in the training set. In each pattern unit, a Gaussian PDF is applied to the network input, so that it is defined as equation (4)

(4) Theta=EXP[−0.5∗(A−t)`(A−t)/(Sigma^2)]

where Theta is the output of the Pattern Unit, A is the origin, t is the vector of training stored in the unit, and Sigma is a positive variable known as the “distance” or “smooth parameter” or ―smoothing factor‖. If Theta is determined, the calculation is transferred to the summation layer P = SUM(P*Theta)/SUM(Theta) where P is the conditional prediction of P and Q is the solution in the sample of training.

### D RBFNN

RBFNN 30 is an artificial neural network that uses radial functions as the activation functions shown in equation (5). RBFNN is a three-layer neural network of feed-forwards. The first layer is linear, transmitting only the input signal, while the next layer is nonlinear, using Gaussian functions. The third layer integrates a linear representation of the Gaussian outputs. Only the tap weights between the hidden layer and the output layer shift during preparation.

(5) $$f(x)=\frac{1}{\sqrt[{\sigma }]{2\pi }}e^{-\frac{{\left(x-\mu \right)}^{2}}{2\sigma^{2}}}$$

### E Nonlinear Autoregressive Neural Network

The Nonlinear autoregressive neural network is a type of ANN that is suitable for estimating future input variable values. The NAR Network helps to forecast future values of the time. It supported the use of a re-feeding mechanism through its historical precedent, in which a predicted value would serve as feedback for new predictions at more advanced points in time. In equation (6) represents as predict series y(t) given d past values of y(t).

(6) y(t)=f(y(t−1),...,y(t−d))

### F Root Mean Squared Error (RMSE)

RMSE is the square root of the square differences measured between predicted and actual COVID-19 Death cases 8. It represents as in equation (7)

(7) $$\mathrm{RMSE}=\sqrt{\frac{1}{n}\sum_{i=1}^{n}(\mathrm{Predicted}\;\mathrm{Death}\;\mathrm{Cas}e_{i}-\mathrm{Actual}\;\mathrm{Death}\;\mathrm{Cas}e_{i}{\text{)}}^{\text{2}}}$$

Where, n= number of samples, Pi=ith Predicted value, Ai= ith Actual value

### G Correlation coefficient (R2)

It measures a linear relationship between the predicted and actual COVID-19 death cases 8. It represents as in equation (8)

(8) $$R=\frac{\sum_{i=1}^{n}\left(a_{i}-\bar{a_{i}})(b_{i}-\bar{b_{i}}\right)}{\sum_{i=1}^{n}{\left(a_{i}-\bar{a_{i}}\right)}^{2}{\sum_{i=1}^{n}\left(b_{i}-\bar{b_{i}}\right)}^{2}}$$

Where is the actual COVID-19 death case value, is the predicted COVID-19 death case value; - is the mean of real COVID-19 death case value - is the predicted mean COVID-19 death cases value, and is the total number of data points.

### Proposed methodology

#### Dataset Description

A dataset has been downloaded from the Kaggle website (www.kaggle.com) for predicting the COVID-19 death cases for India. This dataset contains India's COVID-19 Confirmed cases and Death cases from January 20, 2020, to April 30, 2020, which is used for training and testing models.

First, these data are pre-processed to eliminate missing values and inappropriate values. These data can be used to create two types of datasets. They are:

- Dataset 1 contains two attributes, such as COVID-19 confirmed cases and death cases. Here, ‘death case’ is a predictive attribute and ‘confirmed case’ is a response attribute.
- Dataset 2 contains a time series of COVID-19 death cases.

In this paper, three models (such as GPR, GRNN, and RBFNN) are constructed with the appropriate model parameter values and used in these two datasets to validate the predicted results concerning given the available datasets.

**Figure 1:**
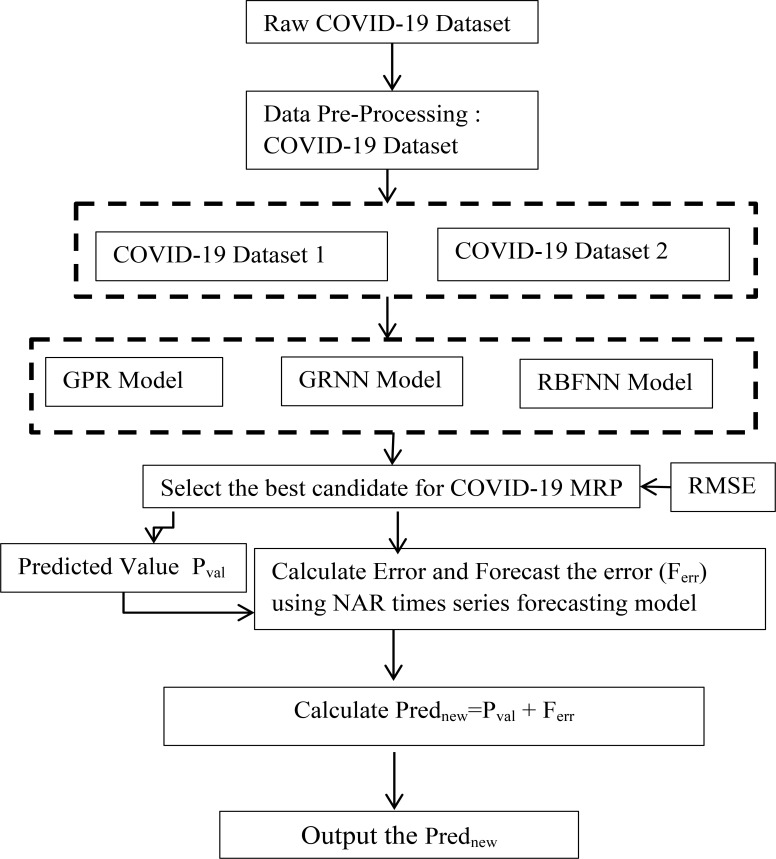
Proposed Methodology for COVID-19 MRP Model

Figure 1 illustrates the proposed methodology. In general, residues or errors are an inevitable part of any predictive or regression models. Similarly, there are errors in the GPR, GRNN, and RBFNN models. To provide a predictive model with high accuracy, this study explores a hybrid approach, including regression methods and the non-linear auto-regression (NAR) neural network (NAR-NN) time series forecasting model. Therefore, trends in residues or errors are detected and predicted by the NAR-NN model. Combining the predicted residual values of each model with the predicted value of each model will provide greater predictive accuracy. The following steps are used to develop the hybrid model:

**Step 1:** Download data from the website and pre-process the dataset. Create Dataset 1(D1) and Dataset2(D2).

**Step 2:** Initialize Model Parameters and define hyperparameters for GPR, GRNN, and RBFNN.

**Step 3:** Input datasets D1 and D2 into the GPR model, GRNN model, and RBFNN model respectively, and predict COVID-19 death cases (Prednew) for ‘n’ period ahead or for given set of confirmed cases.

**Step 4:** The residuals produced by these models are extracted and converted into time-series data.

**Figure 3:**
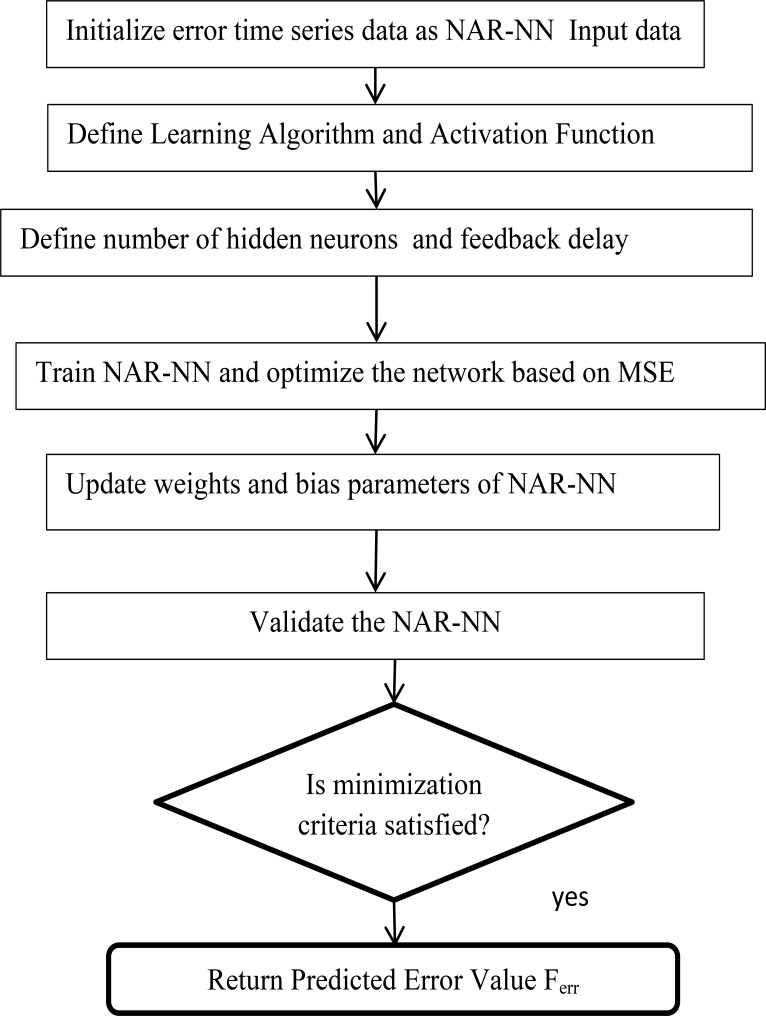
Workflow of NAR-NN Time Series Forecasting

**Step 5:** Input these residuals into the NAR-NN time series forecasting model and predict the residual values (Ferr) for three models separately. It is shown graphically in figure 3.

**Step 6:** Ferr is added with PredNew to generate an optimized prediction value.

**Step 7:** Return optimized predicted values as output

**Figure 2:**
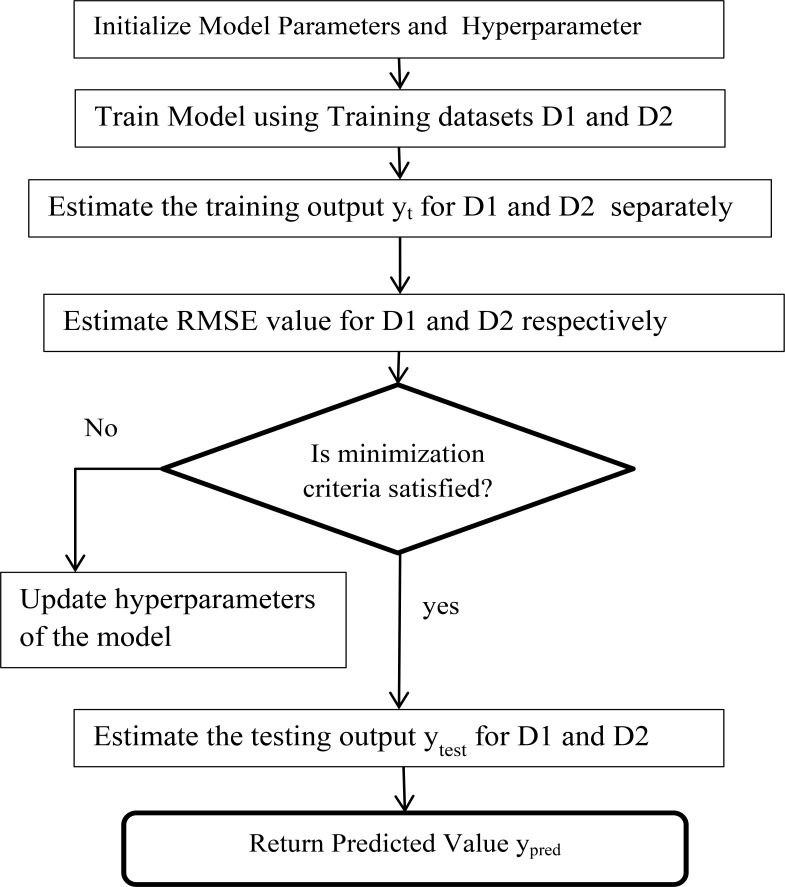
Workflow of Model with Hyperparameter Tuning

Table 1 shows the parameter setup for all three models. Figure 2 illustrates the operating principles of the hyperparameter tuning used with the GPR model, the GRNN model, and the RBFNN model to achieve the minimum RMSE value for these models, respectively. Similarly, figure 3 describes the working principle of the NAR-NN model for error forecasting for these models.

**Table 1:** Model Parameters Setup

Model Parameters	GPR Model	GRNN Model	RBFNN Model	NAR-NN Model
**Hidden Layer (HL)**	N/A	Fixed Architecture	Fixed Architecture	Fixed Architecture
**Number of Neurons in** **HL**	N/A	10	10	15
**Training Algorithm**	N/A	Bayesian Regularization	Bayesian Regularization	Bayesian Regularization
**Kernel Functions**	Exponential	N/A	N/A	N/A
**Hyperparameter**	Covariance (σ) Mean (µ)	Spread/ Smoothing factor (σ)	Spread/ Smoothing factor (σ)	N/A
**Performance**	RMSE	RMSE	RMSE	MSE

## Outcomes and Discussions

This section contains results of Gaussian Process Regression, and two different statistical neural networks (SNNs): GRNN and RBFNN models for dataset1 and dataset^2^ are presented and discussed. The performance of these models was compared.

The benchmark performance metrics such as Root Mean Squared Error (RMSE), an error measure, and Correlation coefficient accuracy measure (R) are used to estimate the COVID-19 death cases prediction accuracy. By using Hyperparameter tuning, the predictive efficiency of these three models can be improved. The goal of hyperparameter tuning is to optimize the value of hyperparameters of each model to minimize the RMSE value of these models. Based on RMSE value, the best model is selected for COVID-19 death cases prediction and mortality rate prediction.

Table 2 shows the values of the performance metrics such as RMSE and R2 for three models. While comparing these models, it is seen that the GPR model has low RMSE value and high R2 value for both datasets. It signifies that the GPR model performs better than two statistical neural networks for the COVID-19 dataset. The spread value (σ) of GRNN and RBFNN is 4 and 1.76, respectively. Table 3 and Table 4 display the values of hyperparameters for statistical neural networks and Gaussian Process Regression models.

**Table 2:** Performance Metrics for Datasets

Model	RMSE_D1	RMSE_D2	R^2^ _D1	R^2^ _D2
**GRNN**	2.44605585	3.249880129	0.999938	0.9999646
**RBFNN**	2.959403436	3.264847469	0.999937	0.999954
**GPR**	0.177218356	0.16828063	1	0.9999998

**Table 3:** Hyperparameter Value for GRNN and RBFNN (Spread)

Dataset	GRNN	RBNN
**D1**	2	1.28
**D2**	4	1.76

**Table 4:** Hyperparameter Value for GPR

Dataset	Sigma M	Sigma F	Sigma
**D1**	6863.8	255	6.8
**D2**	1008.5	79.1	2.6

**Figure 4:**
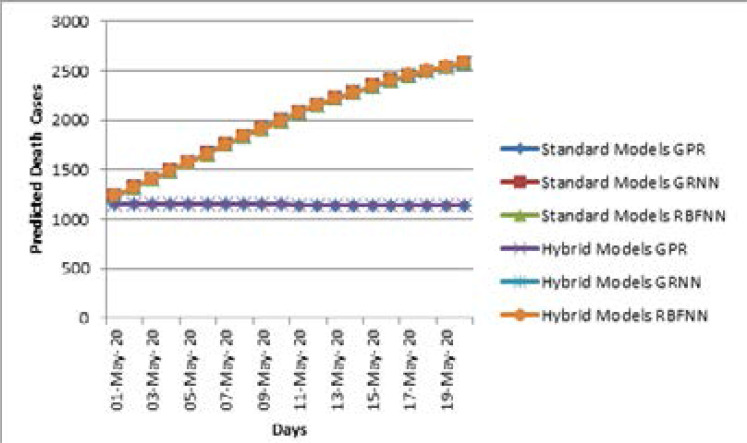
Comparison of Standard and Hybrid Models for D1

Tables 5 and 6 show the predicted number of COVID-19 death cases for all three standard and hybrid models using the time series dataset (i.e. D2). A hybrid model is the combination of standard models and NAR-NN based error forecasting models. There is no difference in the predicted values for the standard and hybrid GPR model since their RMSE value is about 0.2 approximately. Figure 4 shows the number of predicted death cases versus days from May 1, 2020, to May 20, 2020. From this point of view, the two SNNs have nearly equal efficiency, but their RMSE values are higher than that of the GPR models.

**Table 5:** Predicted Value Y_pred_ for Dataset (D2) using Standard Models

Date	GPR	GRNN	RBFNN
**01-May-20**	1152	1234	1234
**02-May-20**	1152	1317	1317
**03-May-20**	1151	1403	1402
**04-May-20**	1151	1490	1489
**05-May-20**	1150	1579	1576
**06-May-20**	1150	1667	1664
**07-May-20**	1149	1754	1750
**08-May-20**	1149	1840	1835
**09-May-20**	1148	1922	1918
**10-May-20**	1148	2002	1997
**11-May-20**	1147	2078	2074
**12-May-20**	1147	2150	2147
**13-May-20**	1146	2218	2216
**14-May-20**	1145	2281	2281
**15-May-20**	1145	2340	2341
**16-May-20**	1144	2395	2398
**17-May-20**	1144	2445	2450
**18-May-20**	1143	2491	2498
**19-May-20**	1143	2533	2543
**20-May-20**	1142	2571	2583

**Table 6:** Predicted Value Y_pred_ for Dataset (D2) using Hybrid Models

Date	GPR	GRNN	RBFNN
**01-May-20**	1152	1233	1233
**02-May-20**	1152	1317	1317
**03-May-20**	1151	1404	1404
**04-May-20**	1151	1490	1488
**05-May-20**	1150	1577	1574
**06-May-20**	1150	1667	1664
**07-May-20**	1149	1757	1751
**08-May-20**	1149	1839	1834
**09-May-20**	1148	1920	1916
**10-May-20**	1148	2003	1999
**11-May-20**	1147	2084	2076
**12-May-20**	1147	2148	2145
**13-May-20**	1146	2220	2213
**14-May-20**	1146	2286	2282
**15-May-20**	1145	2338	2340
**16-May-20**	1144	2395	2396
**17-May-20**	1144	2451	2452
**18-May-20**	1143	2492	2500
**19-May-20**	1143	2534	2540
**20-May-20**	1142	2570	2580

Table 7 shows the predicted number of COVID-19 death cases using the dataset (D1) for three standard and hybrid models. The spread value (σ) of GRNN and RBFNN is 2 and 1.28, respectively.

**Table 7:** Predicted Death Cases for Dataset (D1)

Confirmed cases	Standard Models			Hybrid Models		
	GPR	GRNN	RBFNN	GPR	GRNN	RBFNN
**40000**	1149	1334	1449	1149	1333	1449
**45000**	1145	1458	1659	1148	1458	1665
**50000**	1142	1536	1787	1141	1534	1787
**55000**	1138	1582	1860	1133	1583	1860
**60000**	1134	1610	1898	1133	1609	1898
**65000**	1130	1628	1919	1130	1628	1918
**70000**	1126	1639	1929	1125	1638	1929
**75000**	1122	1647	1935	1122	1647	1935
**80000**	1118	1654	1938	1116	1653	1937
**85000**	1115	1660	1939	1114	1659	1939
**90000**	1111	1665	1940	1109	1663	1940
**100000**	1103	1674	1940	1101	1673	1940

**Figure 5:**
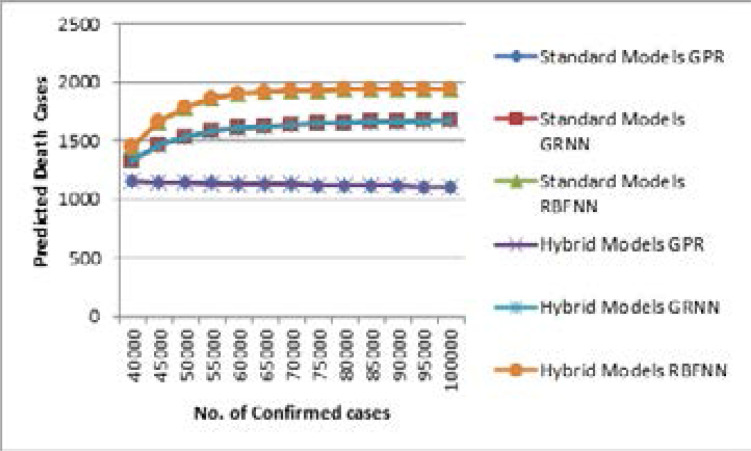
Comparison of Standard and Hybrid Models for D1

From the presented table 7, it is evident that there is no difference in the predicted values for standard and hybrid GPR models using the dataset (D1) since their RMSE value is about 0.18 approximately. Figure 5 shows the number of death cases predicted against the number of COVID-19 confirmed cases. From this perspective, there is no significant difference between the standard and hybrid models for the dataset (D1), as their RMSE values are negligible. However, in this case, the GPR models perform better than the SNNs. Table 8 shows the calculated Mortality Rate Prediction (MRP) for COVID- 19 predicted death cases using the dataset (D1). MRP is defined as equation (9)

(9) $$\text{MRP}=\frac{\mathrm{Number}\;\mathrm{of}\;\mathrm{Predicted}\;\mathrm{Death}\;\mathrm{Cases}}{\mathrm{Number}\;\mathrm{of}\;\mathrm{Confirmed}\;\mathrm{Cases}}*100$$

**Table 8:** Mortality Rate Prediction for Dataset (D1)

Confirmed cases	Standard Models			Hybrid Models		
	GPR	GRNN	RBFNN	GPR	GRNN	RBFNN
**40000**	2.87	3.34	3.62	2.87	3.33	3.62
**45000**	2.54	3.24	3.69	2.55	3.24	3.70
**50000**	2.28	3.07	3.57	2.28	3.07	3.57
**55000**	2.07	2.88	3.38	2.06	2.88	3.38
**60000**	1.89	2.68	3.16	1.89	2.68	3.16
**65000**	1.74	2.50	2.95	1.74	2.50	2.95
**70000**	1.61	2.34	2.76	1.61	2.34	2.76
**75000**	1.50	2.20	2.58	1.50	2.20	2.58
**80000**	1.40	2.07	2.42	1.40	2.07	2.42
**85000**	1.31	1.95	2.28	1.31	1.95	2.28
**90000**	1.23	1.85	2.16	1.23	1.85	2.16
**95000**	1.17	1.76	2.04	1.16	1.76	2.04
**100000**	1.10	1.67	1.94	1.10	1.67	1.94

**Figure 6:**
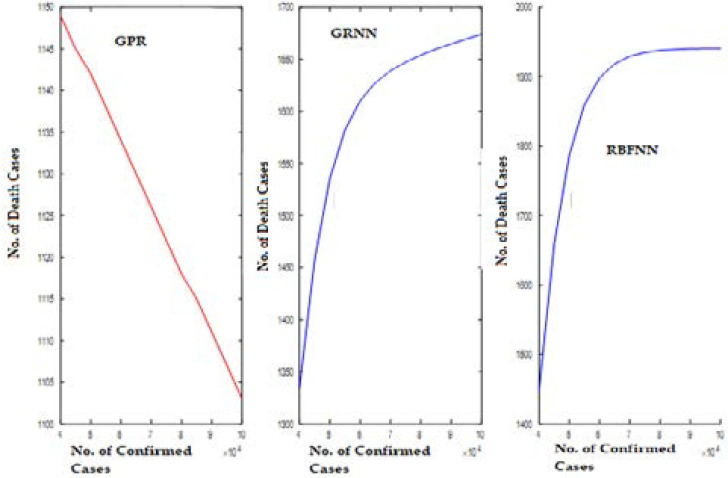
Predicted Curve for Dataset (D1) using GPR, GRNN, and RBFNN

Figure 6 illustrates the predicted curve for COVID-19 death cases versus the number of confirmed cases for India. Here, the X-axis indicates the number of confirmed cases, and Y-axis shows the number of death cases predicted. The GPR model shows a gradual decrease in the number of death cases, while the SNN models show an increasing pattern.

**Figure 7:**
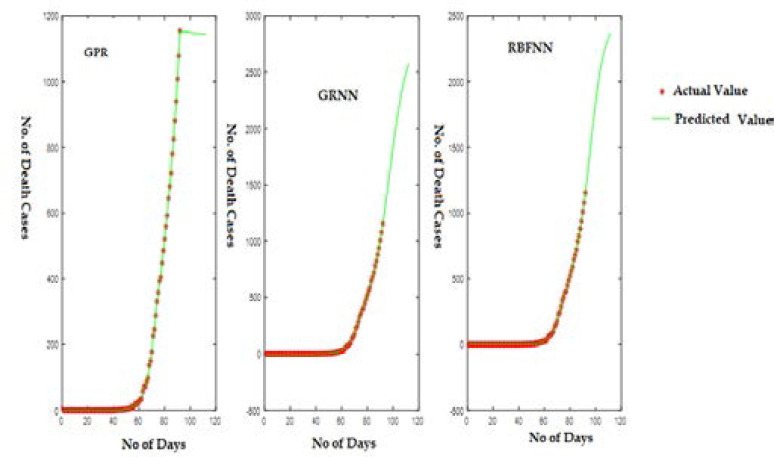
Predicted Curve for Dataset (D2) using GPR, GRNN, and RBFNN

Figure 7 displays the predicted curve for COVID-19 death cases versus the number of days since the first COVID-19 case for India.

Here, the X-axis indicates the number of days, and Y-axis shows the number of death cases predicted. For the dataset (D2) also, the GPR model shows a gradual decrease in the number of death cases, while the SNN models show an increasing pattern.

**Figure 8:**
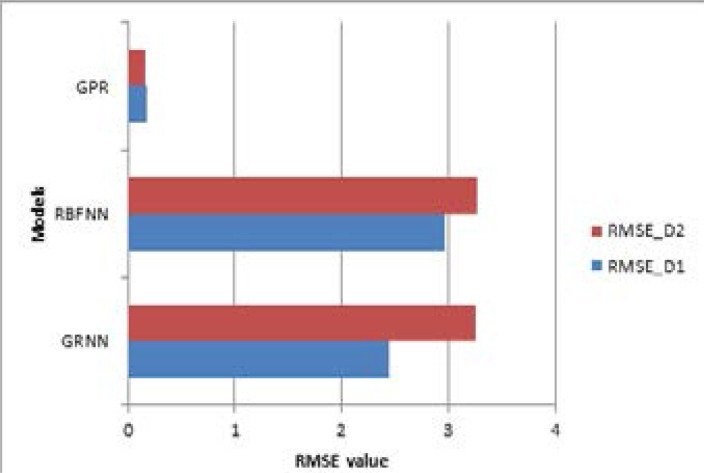
Comparison of RMSE values with a different model

The performance of these models is compared based on RMSE value, as shown in figure 8. From the results, it can be found that GPR performs better than statistical neural networks for two types of COVID-19 dataset.

## Conclusion

This article proposed a methodology that hybridized the regression model (GPR) and the SSN(GRNN and RBFNN) models with the NAR-NN time series forecasting model to achieve higher predictive accuracy in the prediction of COVID-19 death cases. The NARNN time series forecasting model was used to predict errors that should be included in the expected value. The Gaussian Process Regression (GPR) model for the two datasets has yielded a relatively good result in terms of optimized predicted values for death cases in the COVID-19 epidemiological data.

The proposed method is capable of providing a predictive tool for assessing its current state of infection, severity, and help government and health care workers for better decision making to reduce the mortality rate in India.

The first author acknowledges the ―UGC- Special Assistance Programme (SAP) for the financial support to her research under the ―UGC-SAP at the level of DRS-II (Ref.No.F.5-6/2018/DRS-II (SAP-II)), 26 July 2018 in the Department of Computer Science, Periyar University.
